# Antibiofilm Action of Plant Terpenes in *Salmonella* Strains: Potential Inhibitors of the Synthesis of Extracellular Polymeric Substances

**DOI:** 10.3390/pathogens12010035

**Published:** 2022-12-26

**Authors:** Julian J. Palomares-Navarro, Ariadna T. Bernal-Mercado, Gustavo A. González-Aguilar, Luis A. Ortega-Ramirez, Miguel A. Martínez-Téllez, Jesús F. Ayala-Zavala

**Affiliations:** 1 Centro de Investigación en Alimentación y Desarrollo, A. C. (CIAD), Hermosillo 83304, Sonora, Mexico; 2 Departamento de Investigación y Posgrado en Alimentos, Universidad de Sonora, Hermosillo 83000, Sonora, Mexico; 3 Departamento de Ciencias de la Salud, Universidad Estatal de Sonora, San Luis Río Colorado 83500, Sonora, Mexico

**Keywords:** biofilm formation, exopolymeric substances, control biofilm, virulence, terpenoids compounds, foodborne pathogen

## Abstract

*Salmonella* can form biofilms that contribute to its resistance in food processing environments. Biofilms are a dense population of cells that adhere to the surface, creating a matrix composed of extracellular polymeric substances (EPS) consisting mainly of polysaccharides, proteins, and eDNA. Remarkably, the secreted substances, including cellulose, curli, and colanic acid, act as protective barriers for *Salmonella* and contribute to its resistance and persistence when exposed to disinfectants. Conventional treatments are mostly ineffective in controlling this problem; therefore, exploring anti-biofilm molecules that minimize and eradicate *Salmonella* biofilms is required. The evidence indicated that terpenes effectively reduce biofilms and affect their three-dimensional structure due to the decrease in the content of EPS. Specifically, in the case of *Salmonella*, cellulose is an essential component in their biofilms, and its control could be through the inhibition of glycosyltransferase, the enzyme that synthesizes this polymer. The inhibition of polymeric substances secreted by *Salmonella* during biofilm development could be considered a target to reduce its resistance to disinfectants, and terpenes can be regarded as inhibitors of this process. However, more studies are needed to evaluate the effectiveness of these compounds against *Salmonella* enzymes that produce extracellular polymeric substances.

## 1. Introduction

Gastrointestinal infections are caused by consuming contaminated food with enteric pathogens [1]. *Salmonella* is one of the most prevalent causes of foodborne disease and a significant cause of diarrheal illnesses, resulting in 1.35 million infections per year in the United States of America [2,3]. *Salmonella* can sense, adapt, and survive stressful environmental conditions, persisting and resisting disinfection due to their biofilm formation. Biofilms are bacterial communities rounded by a self-produced matrix of EPS [4]. Biofilms have been linked to food product contamination and foodborne illness, causing critical problems in public health [5]. These communities represent a considerable problem for the food industry because the EPS matrix offers protection against the cleaning and disinfection processes, and the contaminated surface could lead to cross-contamination [6].

*Salmonella* can easily adhere to and form biofilms on different abiotic and biotic surfaces [4,7]. *Salmonella* adhesion is the initial stage in biofilm formation; cells can attach to a surface in minutes or hours, depending on environmental conditions [8]. The EPS of the *Salmonella* biofilm matrix have been classified based on their function beyond their composition. Structural EPS represent this classification’s largest and most relevant group in protection against disinfectants. These structural EPS are composed of polymers, such as cellulose, curli, colanic acid, and proteinaceous O-antigen [9].

Biofilms are hard to eradicate because the EPS matrix’s three-dimensional network houses bacteria and protects them from the action of disinfectants [10]. This matrix limits diffusion and inactivates xenobiotic agents inside the biofilm, affecting the disinfection process and compromising food safety. Therefore, recent studies have focused on targeting the formation of the EPS matrix in *Pseudomonas*, *Staphylococcus*, and *Escherichia* [11]. However, this paradigm has been little explored in *Salmonella* [12]. In general, the main components of *Salmonella* biofilms are polysaccharides, such as cellulose, and protein structures, such as curli and adhesive fimbriae; the inhibition of their synthesis could be an interesting target for biofilm control.

For *Salmonella* control, the inappropriate and intensive use of disinfecting agents could induce bacterial resistance and, in some cases, affect food contact surfaces [13]. Therefore, the need to research alternatives for disinfecting agents has been emphasized. Terpenes found in plant sources are a promising alternative due to their effect on bacterial growth, biofilm formation, and enzyme activity [14]. Some studies have reported that terpenes significantly reduce *Salmonella* biofilm formation [15,16,17,18,19]. This effect has been correlated with the decrease in biofilm exopolysaccharide production in *Enterobacter cloacae*, *Staphylococcus aureus*, *Salmonella* Typhimurium*, Escherichia coli* O157:H7*, Listeria monocytogenes*, and *Streptococcus sobrinus* [20,21,22,23,24,25]. However, many details of how this terpene inhibits glucan synthesis are not considered. In other bacterial species, it was observed that terpenes, due to their structure, could potentially interact with glucosyltransferase enzymes that participate in the synthesis of glucans. Ortega-Ramirez et al. [26] showed that citral and geraniol inhibited glycosyltransferase activity, reducing glucans production in *E. coli* biofilms. In contrast, terpenes have also been shown to regulate the expression of genes related to EPS synthesis, such as cellulose, curli, or colanic acid [20].

There is evidence of the efficacy of terpenes in inhibiting EPS synthesis and biofilms of pathogenic bacteria; however, there is still a lack of knowledge of their specific mode of action in *Salmonella* [27]. It is also important to characterize the EPS synthesis during biofilm development and quantify changes in the EPS content under different conditions, such as temperature, nutrients, pH, and contact surfaces, to design more complex and practical systems when exploring the terpenes’ mode of action. In addition, the time-dependent antibiofilm activity of terpenes is not normally studied, and it can be useful to define times of action. Finally, exploring molecular inhibition mechanisms of EPS synthesis at genetic and post-translational levels will generate more solid knowledge of the terpenes’ activity. In this context, this manuscript discusses the importance of *Salmonella* biofilms and the anti-biofilm effect of terpenes targeting the EPS matrix, trying to solve the following research question: How can terpenes affect biofilm formation through inhibiting extracellular polymeric substances synthesis?

## 2. *Salmonella* Is a Risk to Public Health

Diarrhea affects more than 2 billion people globally each year, according to the World Health Organization (WHO). Acute diarrheal conditions are associated with significant mortality and morbidity, and regarding the source of infection, food is involved in a third of the cases. *Salmonella* is a causative pathogen of more than 90 million diseases annually associated with diarrhea worldwide, and salmonellosis is one of the most common foodborne infections [28]. Each year, millions of human infections due to enteric subspecies are reported, including gastroenteritis (non-typhoid salmonellosis) and typhoid fever (Typhi and Paratyphi serotypes). The genus *Salmonella* consists of two species: *bongori* and enterica [29]. The most prevalent causes of outbreaks are *Salmonella enterica* serovar Typhimurium and *Salmonella enterica* serovar Enteritidis [30]. Typhimurium and Enteritidis lead the reports of the 2600 serotypes that cause diarrheal outbreaks; therefore, these subspecies are the main ones responsible for non-typhoid salmonellosis [31].

*Salmonella* is a dangerous pathogen for human and even animal health. The number of infections caused by this microorganism is remarkably high worldwide, and although many are not severe, it leads to hospitalizations and deaths. These infections affect the family economy and impact public health costs due to hospital time, treatment costs, and work productivity loss. The Economic Research Service of the United States Department of Agriculture estimates that the annual cost of foodborne illnesses for *Salmonella* (nontyphoidal) in the United States was USD 4,142,179,160 in 2018 [32]. *Salmonella* disease is a self-limiting infection in healthy individuals; however, older adults, children, pregnant women, and immunocompromised individuals are often more susceptible to severe illness and even death*. Salmonella* cells travel through the stomach and colonize the small and large intestines after consumption. This pathogen infects and multiplies in the gut mucosa and can enter the gastrointestinal tract’s lymphoid tissues and move to circulation. Dissemination to the bloodstream is rare, occurring in fewer than 5% of infections, and depends on the given *Salmonella* strain’s virulence and the host’s immune response [33]. The most typical signs of *Salmonella* infection are fever, diarrhea, and stomach pains [34].

The number of *Salmonella* infection cases is considerably important worldwide (Table 1). According to the Centers for Disease Control and Prevention of the United States of America, *Salmonella* causes roughly 1.35 million infections, 26,500 hospitalizations, and 420 fatalities annually; contaminated food is the primary cause of most diseases. Similarly, the European Food Safety Authority reports 91,857 non-typhoid *Salmonella* infections annually, a major contributor to foodborne outbreaks; it was the second most reported foodborne gastrointestinal infection in humans after campylobacteriosis [3,35,36]. Meanwhile, Mexico reported around 80,000 cases annually [37]. Diagnosed salmonellosis in the EU in 2020 indicated a rate of 13.7 cases per 100,000 people, and the United Kingdom reported 20.4 per 100,000 people. These reports highlight the impact of this bacterium in compromising food safety and indicate its persistence after disinfection procedures. This persistence could be attributed to its capacity to form biofilms on different surfaces, allowing the consequent cross-contamination [38]. Understanding the sources and how *Salmonella* contaminates during these processes is crucial to preventing and controlling infections. Additionally, it is detected that more essential epidemiological studies need to be carried out to identify where and how these infections occur in different regions and food systems. What are the primary contamination sources of *Salmonella*? What are the circulant serotypes and pathotypes? What is their resistance to disinfectants and antibiotics? What is the role of biofilm formation in this resistance?

## 3. *Salmonella* Biofilm Formation Process

**Figure 1:**
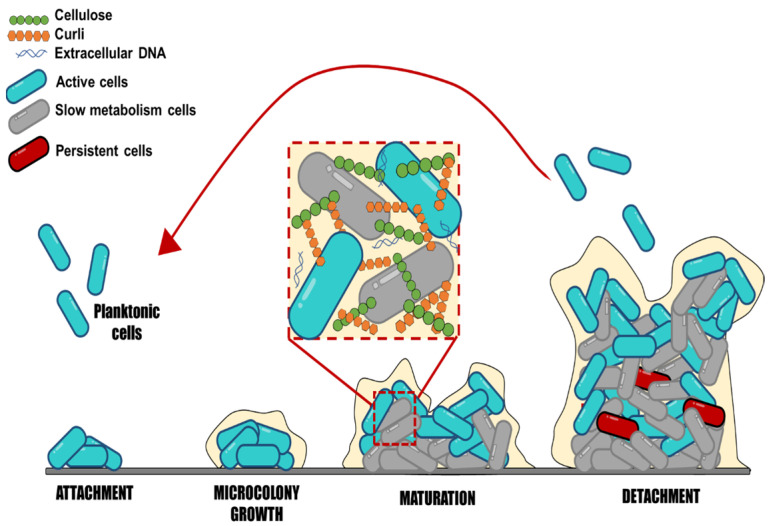
Schematic representation of biofilm formation, step by step. This scheme represents the stages of biofilm development: Attachment, microcolony growth, biofilm maturation, and detachment of embedded cells.

*Salmonella* may produce biofilms. These complex communities adhere to various surfaces, contributing to the bacteria’s resistance and persistence in both host and non-host situations. *Salmonella* biofilm formation is a dynamic and complex process that implies subsequential steps, where sessile cells exhibit a different physiological state than their planktonic counterparts (Figure 1). S*almonella* strains can form biofilms starting with the adherence of planktonic cells to surfaces [6]. The initial cell adhesion to surfaces is influenced by different factors, such as the type of surface, texture (rough or smooth), charge, polarity, pH, temperature, and medium nutrients [6,40].

Moraes et al. [41] showed that the different strains (*S.* Enteritidis, *S.* Infantis, *S*. Typhimurium, and *S.* Heidelberg) have different adhesion abilities and biofilm formation on stainless steel surfaces under different pH, temperature, and NaCl concentrations. All strains demonstrated adherence at pH 4, up to 4% NaCl, and at temperatures of 20 °C and 35 °C. For all studied strains, the chance of adhesion was reduced in conditions where NaCl concentrations reached >8%, 8 °C, and pH 5. Moreover, *Salmonella* can adhere to gallstones, animal epithelial cells, plant surfaces, and abiotic surfaces [8]. Numerous studies have reported that *Salmonella enterica* can adhere and form biofilm on plastic, glass, and stainless steel [42,43,44]. These materials are commonly used in kitchens, toilets, slaughterhouses, farms, and the food industry. A study showed that *Salmonella* adheres differently depending on the surface and temperature conditions, affecting how long they survive in a processing environment [43]. The results showed that at 25 °C, more isolates formed strong and moderate biofilms on plastic surfaces than on stainless steel; at 15 °C, fewer isolates formed strong biofilms than at 25 °C, and plastic surfaces were more prone to adherence than stainless steel at this temperature. The study of *Salmonella* attachment in different conditions may offer important information to design control actions and reduce biofilms in processing environments.

Considering the ability of *Salmonella* to survive on abiotic surfaces, this bacterium can represent a potential danger for consumers by contaminating food products. There is limited comprehension of the effect of surfaces, times, and temperatures used in food industries, as well as the influence of low-nutrient conditions, such as food contact surfaces that may have organic residue on adhesion and biofilm formation of *Salmonella*. It is crucial to describe bacterial adherence, the capacity to form biofilms, and the sanitizer resistance of *Salmonella* to design efficient control measures and hygiene procedures. Some strategies could directly inhibit virulence factors, such as adhesion, EPS secretion, flagella inhibition, and protein synthesis involved in bacterial metabolism or quorum sensing.

*Salmonella* biofilm formation continues with the irreversible adhesion caused by the secretion of EPS composed of polysaccharides, proteins, and DNA, which form the matrix biofilm and increase the cell-surface and cell-cell interactions [4]. The EPS matrix accounts for 90% of the biomass, while microorganism cells contribute the rest, 10%, emphasizing the significance of the EPS matrix. *Salmonella* biofilm’s main components consist of polysaccharides, cellulose, colanic acid, anionic O-antigen capsule, proteins such as the amyloid fibers called curli, flagella, surface protein components, and fatty acids [9]. The amount of each component within *Salmonella* biofilms is still unknown, representing an excellent area for further studies. It is essential to point out that the exact composition of the biofilms cannot be generalized for all cases; it would be interesting to know how the EPS components of the biofilm vary among serotypes or by modifying environmental factors and how this influences disinfection processes. For example, Kim et al. [45] concluded that the optimal condition for total cell mass and EPS synthesis after 9 days of *Salmonella* Typhimurium biofilm maturation was at 15 °C for the rdar (red, dry, and rough) and bdar (brown, dry, and rough) strains compared to 25 and 37 °C. It is necessary to establish the most specific contribution of each type of EPS in biofilm formation, and how this and the environmental factors influence the mechanisms of resistance to and survival of disinfectant processes, because only the contribution of the entire matrix is known.

During biofilm maturation, the EPS matrix creates a three-dimensional network essential to biofilm lifestyle and virulence development; this network protects bacterial cells from environmental stresses, such as antimicrobials and immune system cells. The biofilm biomass provides a hydrated viscous environment that protects cells from various stressors, including desiccation, disinfectants, antibiotics, temperature, and oxygen content [46]. It may also prevent the loss of enzymes, nutrients, and molecules that could favor the microenvironment for bacteria within the biofilm [47]. However, the lack of knowledge detected on this point is regarding biofilm characteristics to consider its maturity level; these characteristics may involve measuring the number of adhered cells, EPS content, and variations among serotypes at different times. *Salmonella*’s lifetime is finished when cells leave, disperse from the biofilm, and revert to planktonic mode [48]. The dispersion of bacteria in biofilms should also be explored to a greater extent, because once they are released from the biofilm, they can colonize new sites and persist in the medium, making disinfection processes more difficult. Still, limited information is available on the time at which biofilm dispersal begins, as well as what environmental factors promote it.

Structural EPS is the largest and most relevant group of substances that interferes in bacterial disinfection tasks. They primarily consist of neutral polysaccharides and protein parts that aid construction and surface colonization [49]. These EPS contribute to the formation processes, highlighting cellulose as one of the main components, followed by other components, such as curli, cholic acid, and protein O antigen material [9]. For all the stated above, biofilms are extremely difficult to remove from surfaces in the food industry. A lack of knowledge is detected around the potential variations in biofilm development, composition, and resistance among *Salmonella* strains with different morphotypes. In this context, it will also be interesting to determine the ideal conditions for biofilm formation and development.

## 4. Main Structural EPS Produced during *Salmonella* Biofilm Formation

The main components of *Salmonella* biofilms are polysaccharides, such as cellulose, and protein structures, such as curli. This section describes the primary structural EPS involved in *Salmonella* biofilm.

### 4.1. Curli

**Figure 2:**
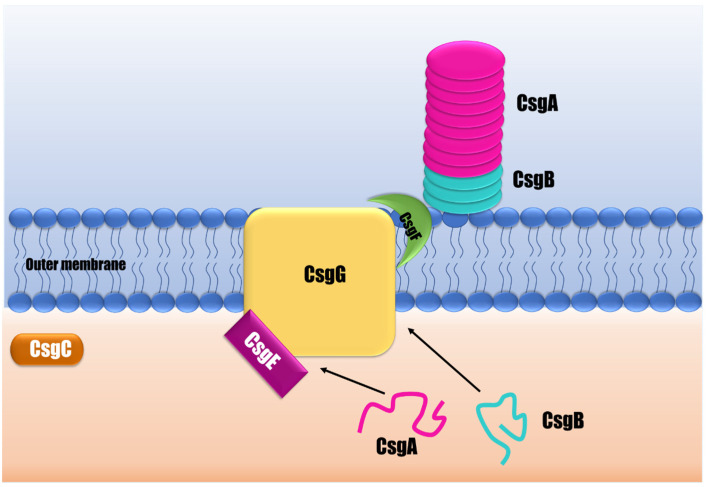
A schematic representation of curli biogenesis. The curli assembly mechanism is an outer membrane secretion apparatus characterized by exporting CsgA subunits that will precipitate in the presence of a nucleator that fixes the fibril on the bacterial surface. The CsgG is a transmembrane pore responsible for the secretion of soluble CsgA in the extracellular medium, helped by CsgE. CsgG directly interacts with CsgF to translocate the nucleator CsgB and contributes to the assembly of an amyloid conformation. CsgA interacts with CsgB, and they assemble into an amyloid fiber. In contrast, CsgC is an oxidoreductase with an unknown specific activity.

Curli are amyloid fibers that make up most of the *Salmonella* biofilm’s matrix protein fraction [50]. Curli biogenesis is a complex and highly controlled process that demands many proteins to synthesize a functional fiber [50]. This EPS is produced by a multicomponent secretion system that facilitates the transit of curli subunits between the periplasm and outer cell membrane, and controls the self-assembly of curli subunits into fibers attached to bacterial surfaces (Figure 2) [51]. In *Salmonella*, curli are encoded by two separate operons: *csgBAC* and *csgDEFG* [52]. The *csgBAC* encodes for the major subunit CsgA and the minor subunit CsgB. CsgA structurally constitutes the fibers, but firstly is released as an unstructured soluble peptide over the outer membrane. Then the nucleator and minor curli component CsgB combines to create the amyloid fiber on the cell surface [53,54]. Both subunits, CsgA and CsgB, have a similar structure composed of three domains: a signal peptide, an N-terminal segment, and a C-terminal amyloid core domain consisting of five repeating domains able to form beta-sheet structures [55]. The third protein encoded by *csgBAC* is the CsgC, a periplasmic protein that seems to act as a highly effective inhibitor of CsgA amyloid formation in order to prevent toxic intracellular aggregates [53].

The subunits encoded by *csgDEFG* form the curli secretion system comprising at least three proteins: CsgG, CsgE, and CsgF. CsgG is a lipoprotein forming a nonameric secretion channel that traverses the outer membrane to transport CsgA and CsgB from the periplasm to the extracellular environment [56]. CsgE is a periplasmic protein that plays an assisting function, interacting with curli subunits (CsgA) targeting the CsgG channel for secretion [55,57]. CsgF is a surface-exposed protein required for specific localization and chaperoning of the nucleating activity of CsgB to form functional fiber subunits [58]. The lack of these proteins results in defective curli assembly [56,57,58,59].

CsgD is a transcriptional regulator in *Salmonella enterica* and the major regulator in biofilm formation and curli synthesis. CsgD regulates the expression of *csgBAC,* promoting the transcription of the CsgA and CsgB structural components of curli fiber [60]. The activation of CsgD in *Salmonella* is highly regulated by global transcriptional regulators, such as RpoS, EnvZ/OmpR, CpxA/CpxR, and H-NS, which mediate diverse cellular responses as a function of changes in the environment. Minimal osmolarity, low temperatures, and restricted nutrition are required to activate csgD transcription [61]. Van Gerven et al. [55] and Evans and Chapman [53] have conducted more in-depth and extensive reviews on fimbria curli’s structure, biogenesis, and regulatory mechanisms.

There is still information that is not clear on the synthesis and regulation of fimbria curli, such as what environmental factors stimulate CsgD for fimbria synthesis and whether the production of fimbria curli is temperature- or strain-dependent, as temperatures as low as 30 °C promote curli expression, but biofilm formation has also been observed at higher temperatures. It is also necessary to explore the structures of Csg proteins involved in curli synthesis and the genetic activity of two operons (*csgBAC* and *csgDEFG)* to determine if plant terpenes could modify their expression and activity to combat curli-dependent biofilms.

### 4.2. Cellulose

Cellulose is a long polymer of β-(1→4)-linked D-glucose units, and it is the second most important component in *Salmonella* matrix biofilms [46]. The cellulose polymerization and translocation processes begin with binding glucose-1-phosphate with UTP (uridine-5′-triphosphate), forming UDP-glucose in the intracellular glucosyltransferase domain. In the second step, the UDP-glucose is relocated to the 4′-OH group at the non-reduced terminal end of the polymer chain, expanding it and releasing the UDP. In the third step, the polymer must be translocated into the transmembrane channel to allow the addition of a new glucose molecule [62]. The *bcs* (bacterial cellulose synthesis) operon contains four genes, *bcsA*, *bcsB*, *bcsC*, and *bcsD*, that are required for cellulose production and secretion [52].

**Figure 3:**
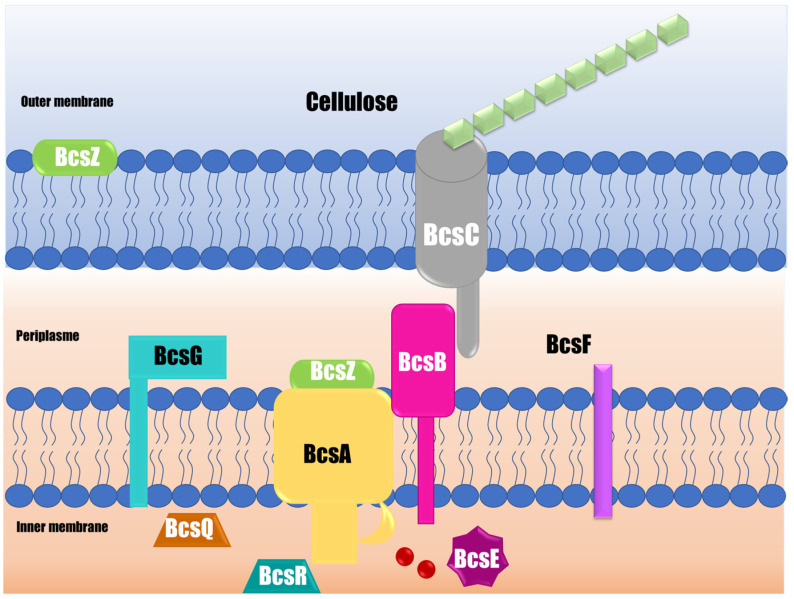
Scheme of the cellulose synthase complex responsible for cellulose synthesis in *S*. Typhimurium. BcsA produces glucan chains by glycosylating UDP-glucose; BcsB forms complexes with BcsA to activate cellulose synthase; and BcsC creates a channel in the outer membrane, through which glucan chains are extruded. BcsE, BcsF, BcsG, BcsQ, and BcsR participate in cellulose synthesis with different and unknown functions.

The polymer is synthesized by a complex of three transmembrane glucosyltransferases (BcsA, BcsB, and BcsC subunits) (Figure 3). BcsA is the catalytic component synthesizing cellulose and creating the inner membrane transmembrane pore [63]. This BcsA subunit contains eight transmembrane fragments and, at minimum, one glucosyltransferase -A-folded intracellular extended domain. The glucosyltransferase -A intracellular domain oversees binding the donor sugar and the acceptor, in addition to activating the glucosyltransferase reaction and the part immersed in the membrane to form a pore next to the catalytic site, allowing the nascent polysaccharide to be translocated [62]. It is important to point out that this cellulose synthase scheme was developed for a *Rhodobacter* model. Currently, no evidence of cellulose synthesis proteins and crystallized proteins have been found in the case of *Salmonella*. It has been reported that the catalytic site is well conserved and contains the signature aspartic acid, aspartic acid, aspartic acid, glutamine (glutamine/arginine), X (any amino acid), arginine, tryptophan of three variably spaced aspartic acids, and a pentapeptide consisting of glutamine that is often followed by a glutamine or arginine, a variable residue, arginine, and tryptophan. However, there may be allosteric sites that allow modifying the activity of the enzyme. The BcsB subunit is a large periplasmic protein that can direct the polymer through the periplasm to the outer membrane through two carbohydrate-binding domains [52]. Although BcsA is the catalytically active component, catalysis requires the membrane-anchored BcsB subunit. BcsC, for its part, creates a β-barrel in the outer membrane, which is preceded by a large periplasmic domain that includes a tetratricopeptide, likely participating in the building of the complex and comprising the outer membrane pore [63].

Other proteins also participate in cellulose synthesis and are encoded by the bcsABZC and bcsEFG operons [46]. The BcsZ is a periplasmic protein whose function is not widely known, although it improves cellulose production. Ahmad et al. [64] demonstrated that *S.* Typhimurium BcsZ negatively regulates cellulose synthesis, which affects biofilm formation, host interaction, and organ colonization in the infection of a murine model. Other proteins, such as BcsE, BcsF, BcsG, BcsQ, and BcsR, also participate in cellulose synthesis, although their function is not fully clarified [65]. The transmembrane portion of BcsG is necessary for the BcsA subunit to be produced correctly [65]. The di-guanylate cyclase AdrA produces the c-di-GMP needed to activate cellulose biosynthesis. The CsgD response regulator, which has a favorable effect on adrA, regulates its activity at the transcriptional level [66]. CsgD, in its non-phosphorylated form, binds to the adrA promoter in a complicated way, and AdrA controls bcsABZC by changing the amount of c-di-GMP [64]. There is limited information about the structure of proteins related to cellulose synthesis, such as *Salmonella* glucosyltransferase. Furthermore, it is necessary to study the regulation of cellulose synthesis and how it is influenced by external factors, such as time, temperature, nutrients, and surface used in food processing industries. The generation of this knowledge could allow the development of strategies that inhibit cellulose synthesis and, as a result, have more susceptible biofilms.

### 4.3. Colanic Acid

Colanic acid is likewise categorized as structural EPS. The colanic acid comprises repeated subunits of D-glucose, L-fucose, D-galactose, and D-glucuronic acid sugars with O-acetyl and pyruvate side chains; it is made in a similar way as lipopolysaccharide O-antigen [67]. The tolerance of *S. enterica* to desiccation on a leaf surface is linked to colonic acid biosynthesis [68]. In lysogeny broth (LB), colanic acid was required to create a tight pellicle (biofilm production at the water/air interphase) [69], and its involvement in attachment was assessed using a *wcaJ* insertional mutant [70]. Colanic acid synthesis requires an undecaprenylphosphate glucose phosphotransferase encoded by *wcaJ*. In opposition to cellulose, alfalfa sprout adhesion and colonization were unaffected when the *S. enterica wcaJ* deficient mutant in colonic acid was evaluated [70]. Plant tissue has distinct needs for attachment and colonization compared to animal tissue; hence structural EPS may be controlled differently. Colanic acid does not appear necessary to produce *Salmonella* biofilms on gallstone surfaces (utilizing a *wcaA* mutant) [46].

Other structural EPS components produced by *S.* Enteritidis vary depending on the strain. Furthermore, a novel structural-branched tetrasaccharide EPS has been discovered in *S. enterica* serovar Enteritidis 27655-3b biofilm that differs from cellulose and colanic acid [71]. The authors postulated the structure of this polysaccharide using nuclear magnetic resonance spectroscopy. The repeating unit of this primary saccharide is a branched tetrasaccharide with the following structure: 3)—D-Galp- (12) -[α-Tyvp-(1→3)] -α-D-Manp-(1→4) -L-Rhap-(1-L-Rhap-(1-L-Rhap-(1-L-Rhap-(1-L) With a glucose-containing side chain, this molecule is largely replaced on both tyvelose and galactose. It also has several similarities to the O-antigen, which is found in many different Gram-negative bacteria. A lipid anchor has also been discovered [59,71].

This section only described the primary EPS detailed in the literature for *Salmonella* biofilms. These EPS are essential for biofilm structure and contribute to disinfectant resistance. There are still many unknowns about the synthesis of EPS and its relationship with the biofilm, such as the ideal conditions for the synthesis of EPS in the food industry, the differences in the EPS synthesis processes according to different strains, the contribution of each EPS component in the biofilm, and the resistance and persistence of biofilms. In addition to knowing the structures of the proteins related to these processes and their regulatory mechanisms, seek control strategies, such as the design of molecules that inhibit specific parts of the EPS synthesis process or decrease their gene expression.

## 5. Inhibition of Extracellular Polymeric Substances to Reduce Biofilm Formation

The traditional process to eradicate bacterial biofilms on food contact surfaces is cleaning and disinfection with conventional chemicals, such as chlorine, peracetic acid, and sodium hypochlorite [72]. However, current sanitation methods in the food industry have some well-known disadvantages, such as toxic residues of disinfection agents, corroded food contact surfaces, or the increasing resistance of these chemicals in microorganisms transmitted by food [73].

The EPS matrix facilitates surface adhesion and colonization; therefore, the inhibition or disruption of EPS could be viewed as a target to prevent or eliminate biofilms. A promising concept to fight against biofilm infection is agents that could attenuate the production of those structural components inside the EPS matrix. Powell et al. [11] postulated that *Pseudomonas aeruginosa* preformed biofilms treated with oligosaccharides caused a significant decrease in the fluorescence intensity of ConA and TOTO-1 staining, indicating a biofilm disruption through the reduction in polysaccharides and extracellular DNA components of the matrix biofilm. Although the composition of *Pseudomonas* biofilms is different compared to *Salmonella* biofilms, the paradigm of targeting EPS and disrupting biofilms in *Salmonella* needs to be explored. Extracellular DNA (eDNA) has recently been discovered to be a novel element of *Salmonella* biofilms. eDNA was found and described in *S. enterica* ser. Typhi ST6 and 2-day-old *S.* Typhimurium SR-11 biofilms. According to reports, the eDNA acts as a biofouling agent. It is important to emphasize that the role of eDNA as an informative EPS component is not entirely established. More research must be done to fully comprehend how horizontal transmission could affect the development of biofilms’ development. As an alternative, the eDNA could help provide structural functions [74].

It has been reported that curli-deficient *S. enterica* strains isolated from produce, meat, or clinical sources are the least effective in biofilm formation [75]. Moreover, curli is involved in *Salmonella* invasion, colonization, and persistent infections. The csgA gene’s deletion in *S. enterica* serovar Pullorum strain S6702 decreased curli production and biofilm formation, and reduced adhesion and invasion to HeLa cells’ intracellular proliferation in HD11 macrophages [27]. González et al. [76] reported that curli fimbriae are highly induced in a simulated human gallstone environment by developing biofilms on cholesterol-coated surfaces when bile was present. In a similar context, Adcox et al. [77] suggested a more significant contribution of curli in biofilm formation and gallbladder colonization than other extracellular matrix components.

In contrast, curli is also crucial for attachment to plant surfaces. For example, curli was essential for transferring or surviving *S.* Typhimurium in parsley plants from contaminated irrigation water [78]. Brankatschk et al. [79] examined the whole transcriptome of *Salmonella* Weltevreden using RNA-seq and revealed that genes involved in the curli assembly were upregulated in the presence of alfalfa sprouts, suggesting an essential factor in colonization.

Understanding curli fibers’ complex synthesis mechanism and their components allow the design of inhibition strategies against biofilm-related infections. For example, Yan et al. [80] used the dual-pore architecture of the CsgF-CsgG complex to create a peptide that physically reduces the size of the CsgF pore-inhibiting curli secretion and, thereby, curli amyloid fiber production in *E. coli*. Similar approaches have focused on preventing CsgA fiber polymerization. These studies demonstrated the activity of these treatments; however, there are still unknowns to be resolved and deepened. For example, some of these studies are in vitro, and evaluating more food systems or food contact surfaces would help us better understand biofilms’ behavior.

Several studies have established that cellulose is involved in *Salmonella* biofilm formation and is essential for boosting resistance, which helps bacteria survive [45]. Yaron et al. [81] reported that cellulose of *S. enterica* is an adherence factor in plant materials. Bhowmick et al. [82] reported that deletion of *gcpA* in *Salmonella enterica* serovar Weltevreden caused the inability to produce cellulose. Consequently, the bacteria could not bind calcofluor to grow rdar colony on Congo Red-agar plates and develop biofilms on polystyrene surfaces.

El Hag et al. [27] reported that deleting *csgA* and *bcsA* (deficient for curli and cellulose production) reduced biofilm formation in *Salmonella* Pullorum strain S60702 in glass tubes. However, Δ*csgA* strain bacteria lowered adhesion and invasion into HeLa cells, but Δ*bcsA* did not. As cellulose is a crucial component of the biofilm matrix, inhibiting its production can be considered a control point for *S. enterica* biofilms. It is necessary to generate more knowledge regarding the terpenes’ effect on the genetic expression that encodes cellulose synthase. Similarly, post-translational studies are needed to understand the inhibition mechanisms of this enzymatic system; however, crystallizations of these proteins and structural characterization are required. Therefore, this enzyme could be a control point to inhibit cellulose synthesis and biofilm formation.

As reviewed in this section, curli and cellulose are essential for *Salmonella* biofilms on different surfaces. Moreover, curli and cellulose-dependent biofilm are linked with several infections, including gastroenteritis [83]. Hence, developing agents to combat curli- and cellulose-dependent biofilms is a critical and pressing challenge. Studies suggested that EPS synthesis could be a biofilm control point, as the decrease in its content produces weaker and less structured biofilms, making them less resistant to conventional disinfectants. The identified knowledge gaps open a large area to explore agents that inhibit EPS synthesis and control biofilms in the food industry.

## 6. Terpenes as Potential EPS Inhibitors in *Salmonella* Biofilms

A promising alternative to prevent bacterial biofilm formation is the application of natural antimicrobials [8]. Terpenes and terpenoids, such as thymol, carvacrol, eugenol, and menthol, contained in essential oils, exhibit pronounced activities against diverse microorganisms (Table 2). Several essential oils and their main compounds have exerted antibiofilm activity against *Salmonella* [84]. For example, clove essential oil at 1.2 mg/mL decreased to 1.8 log CFU/cm^2^ of attached *S.* Typhimurium on stainless steel [15]. Carvacrol and thymol, phenolic components in oregano and thyme essential oils, exhibited antibacterial and antibiofilm action against *S*. Typhimurium [16]. Young and mature biofilms produced by *S.* Enteritidis on stainless steel surfaces were destroyed by essential oils of *Origanum vulgare* (0.25%) and *Rosmarinus officinalis* (4%), demonstrating a time-dependent impact and multitarget action mechanism on the bacterial membrane [17]. Čabarkapa et al. [19] investigated the antimicrobial and antibiofilm activities of *Origanum vulgare*, *Origanum heracleoticum*, *Thymol vulgaris*, and *Thymol serpylluma* versus *S*. Enteritidis. The results demonstrated that the essential oils and their principal constituents (carvacrol and thymol) prevented biofilm development at sub-MICs levels and eradicated 48 h formed biofilms over time in a concentration-dependent approach. Cinnamaldehyde (2 mg/mL), the major compound of cinnamon bark essential oil (55–76%), reduced the initial biofilm population by 6 log CFU/cm^2^ of *Salmonella* isolated from a conventional swine farm environment [85].

However, few investigations on *Salmonella* have explored terpenes’ mechanisms to inhibit biofilms’ formation and eradicate them. Some studies in Table 2 have shown that the impairment of EPS synthesis is one of the mechanisms of phenolic compounds and terpenes in other bacteria. For example, carvacrol at 1.33 mM reduced the biofilm formation of *Pectobacterium carotovorum*—a bacterium that causes soft rot in plant food, attributed to its effect on EPS synthesis—for 24 h [88]. This compound at 0.66 mM (sub-inhibitory concentration) reduced the polysaccharides content in *P. carotovorum* biofilms from 22.94 to 9.11 glucose equivalent/cm^2^. At the same time, protein content was reduced, but to a lesser extent, from 11.07 to 7.10 albumin equivalent/cm^2^ [88]. Similarly, carvacrol at sub-inhibitory doses (64 and 128 μg/mL) reduced the biofilm formation of *Enterobacter cloacae* [20]. This effect was correlated with a decrease in biofilm exopolysaccharide production, which was observed in lower biofilm thickness and extra polymeric matrix by confocal laser scanning microscopy and scanning electron microscopy studies. In addition, the transcriptional study demonstrated that carvacrol down-regulated some genes, including curli fimbria and colonic acid polysaccharides necessary for biofilm formation in *E. cloacae* [20].

In the same field, pimento berry, clove, and bay essential oils, and their main frequent component eugenol (0.0053 mg/mL), decreased the enterohemorrhagic *E. coli* O157:H7, an important foodborne pathogen worldwide [21]. The same study indicated that the eugenol structure, including the C-4 alkyl or alkane chain on the benzene ring, the C-2 methoxy unit, and the C-1 hydroxyl unit, have an essential function in antibiofilm mechanisms. This research also used transcriptional analysis to find that eugenol down-regulated some genes, such as fimbria type 1, fimbria curli, and cellulose (*csgABDFG*), in *E. coli*. The reviewed evidence indicated that plant terpenes affected EPS-secreting proteins in Gram-negative bacteria similar to the systems in *Salmonella*; therefore, we could hypothesize a similar effect.

Hakimi Alni et al. [22] found that *Cuminum cyminum* essential oil, whose main compound is the terpenes terpineol, carene, and pinene, inhibited the growth of *S.* Typhimurium at 2.62 μL/mL. This essential oil at sub-inhibitory concentrations also reduced biofilm formation, and it caused the release of cells from biofilms, compared to control bacteria clinging together tightly and encased in a thick matrix. Moreover, these authors reported that the sub-inhibitory concentration of *C. cyminum* oil down-regulated significant genes related to biofilm formation, such as cellulose synthesis (*csgD* and *adrA*) and *Quorum sensing* (*sdiA* and *luxS*) genes.

The clove essential oil, whose main component is eugenol, affected *L. monocytogenes* biofilm formation and removed biofilms on vegetable surfaces [24]. The results showed that all tested concentrations (0.5, 1.0, and 2.0 mg/mL) decreased 35–88% of extracellular polysaccharides and 34–76% of protein content in *Listeria* biofilms. In addition, this essential oil regulated the expression amounts of genes *agrA*, *agrD*, *agrC*, and *prfA*, and it upregulated the expression intensity of the gene *sigB*, thus controlling the formation of biofilms.

Similarly, Li et al. [25] determined the effect of eugenol on glucan synthesis and biofilm of *Streptococcus sobrinus*, a caries-related pathogen. The results showed that an artificial mouth model’s minimum biofilm inhibitory concentration of eugenol (8 mg/mL) was 0.625 mg/mL. The reduction of insoluble glucan synthesis was 63% and 46% for soluble glucans. It is important to highlight that glucans are crucial for the biofilm of caries bacteria. However, many details of how this terpene inhibits glucan synthesis are not mentioned. Eugenol could potentially inhibit some enzymes in synthesizing glucans, such as glucosyltransferase. In addition, several studies conducted in this approach use oral bacteria, thus it would be essential to carry out additional studies that could elucidate the mechanism of glycosyltransferases in food pathogens.

In this sense, Ortega-Ramirez et al. [26] found that citral and geraniol terpenes present in the *Cymbopogon citratus* essential oil inhibited planktonic growth (1.0 and 3.0 mg/mL) and biofilm formation of *E. coli* (2.0 and 4.0 mg/mL) on stainless steel. The mode of action of biofilm inhibition was attributed to the decrease in glucans production; citral and geraniol inhibited glucosyltransferase activity, demonstrating IC_50_ of 8.5 and 6.5 µM, respectively. Molecular docking showed that the most likely interactions between the terpenes and the enzyme occurred inside the hydrophobic pocket under the activation loop and near the enzyme’s finger helix. Terpenes modified glycosyltransferase activity based on the kinetic constants obtained; this suggested a mechanism of non-competitive inhibition of glycosyltransferase by citral and geraniol [26]. The terpenes’ interaction inside the hydrophobic bag beneath the activation circuit and the finger helix may influence UDP-glucose binding and glucan synthesis [89]. These authors describe a possible mechanism employing computational analysis of molecular couplings; therefore, complementing future studies, such as circular dichroism, RMN, and X-ray diffraction methods, could discover the mechanisms of action.

Carrying out a brief analysis of the physicochemical characteristics of some terpenes, such as eugenol, carvacrol, and thymol, which are primarily a function of their structure that has an aromatic ring, it is suggested that this hydrophobic nature gives advantages to it to diffuse through the bacterial membrane in order to reach the intercellular space [90]. In addition, this molecule contains a polar part due to its hydroxyl group; this could allow it to interact with amino acids from the active site and modify the enzymatic action of glucosyltransferase. Once this molecule is in the intercellular region, it could interact with the catalytic subunit BcsA, which contains polar amino acids, such as aspartic acid, glutamine, or arginine, in the conserved domain [62]. These interactions could be by hydrogen bonds of the OH group of eugenol, with oxygen from the side chain of polar amino acids. However, the evidence that has been found to this day does not seem sufficient to decide what type of inhibition could occur and at which site of the enzyme; therefore, studies with this molecule and glucosyltransferase could clarify the information already mentioned above.

In contrast to the inhibition of the cellulose-synthesizing enzyme, most studies evaluating curli synthesis inhibition are associated with terpenes’ effect on the gene expression of curli-related proteins. However, it is still necessary to delve into the mechanism to achieve this reduction. Furthermore, it would be enriching to research the exploration of the protein structures related to the curli, such as CsgA, CsgB, and CsgC, to design agents that possibly affect the assembly of fimbria amyloid. In addition, further studies need to evaluate whether terpenes directly influence these proteins, and how this affects adhesion to different surfaces and biofilm formation. This review indicates the lack of studies that demonstrate the potential of terpenes on the specific inhibition of colanic acid; the studies carried out evaluate EPS in a general way, and few studied the effect on each component of the biofilm.

## 7. Conclusions and Future Perspectives

The present review highlights biofilm formation as an important virulence factor of *Salmonella*; additionally, some structural components, such as cellulose, colanic acid, and curli, are the main structural components of the EPS that cause persistence against disinfection procedures. Therefore, it seems feasible to visualize these virulence factors as a control point. Terpenes are an anti-EPS option, considering their ability to penetrate bacterial cells; these molecules are hydrophobic, and some have hydroxyl groups; these characteristics could inactivate enzymes involved in EPS synthesis in *Salmonella*. Moreover, terpenes can inhibit EPS synthesis due to interference with the expression of cellulose or curli genes. Therefore, future studies to elucidate the characteristics, structure, and allosteric sites of enzymes and genes involved in EPS synthesis are necessary to verify the capacity of terpenes to show how these molecules inhibit different virulence factors and biofilms. In addition, deepening this knowledge would allow us to develop new disinfection technologies and even combine these with some other treatments.

## Figures and Tables

**Table 1:** Cases of people infected with *Salmonella* Typhimurium around the world.

Number of Cases	Bacteria	Source of Contamination	Country	Year
9	*Salmonella* Thomson, Oraniengbur, Typhimurium, Weltevrede, Enteritidis, Hadar	Salami sticks, seafood, onions, Italian-style meats, prepackaged salads, frozen cooked shrimp, raw froze breaded stuffed chicken products, cashew brie, and ground turkey	USA	2021
473	*Salmonella* Braenderup, Muenchen, Thompson, and Typhimurium	Various	USA	2020
1000	*Salmonella* spp.	Backyard poultry	USA	2019
80	*S.* Typhimurium	Sushi	Chile	2019
49	*Salmonella* spp.	Chicken-sandwich products	Australia	2018
40	*Salmonella* Concord	Tahini products	Israel	2018
14	*S.* Typhimurium	Dehydrated coconut	USA	2018
265	*S.* Typhimurium	Chicken salad	USA	2018
87	*Salmonella* spp.	Unknown	Japan	2017
24	*S.* Typhimurium	Strains used for educational purposes	USA	2017
907	*Salmonella* Poona serotype	Cucumber	USA	2016
230	*S.* Typhimurium	Raw mung bean sprouts	South Australia	2016
44	*S.* Typhimurium	Raw breaded chicken	Canada	2015
41	*S.* Typhimurium	Strains used for educational purposes	USA	2014
22	*S.* Typhimurium	Ground beef	USA	2013
261	*S.* Typhimurium	Cantaloupe melon	USA	2012

Source: Centers for Disease Control and Prevention (CDC) of United States of America [31], Popa*,* et al. [39].

**Table 2:** Antibacterial efficacy and mode of action of different terpene compounds against food pathogenic bacteria.

Compound	Concentration Used against Bacteria	Mechanism Action	References
Eugenol	*E. coli* (800–3000 μg/mL) *L. monocytogenes* (800–1000 μg/mL) *S. enterica* serovar Thypimurium (3.18–500 μg/mL)	Inhibition: ATPase, histidine decarboxylase, and extracellular enzyme production (at sublethal concentrations) Membrane permeability ATP and potassium ion leakage	[86]
Eugenol	IC_50_ (µM) 97.31	Deoxy-D-xylulose 5-phosphate reductoisomerase interaction with amino acids Lys124, Asp149, Ser150, Trp211, Met213, Ile217, Glu230 and Met275 (hydrogen bond with Asn226) Competitive inhibition	[87]
Carvacrol	IC_50_ (µM) 139.24	Deoxy-D-xylulose 5-phosphate reductoisomerase interaction with amino acids Trp211, Ser212, Met213, Asp274 and Met275 (hydrogen bond with Pro273) Uncompetitive inhibition	[87]
Eugenol	0.625 mg mL^−1^ CMI	Inhibition of glucan synthesis by *Streptococcus sobrinus*	[25]
Citral	IC_50_ 8.5 µM *E. coli*	Non-competitive inhibition	[26]
Geraniol	IC_50_ 6.5 µM *E. coli*	Non-competitive inhibition	[26]
Cinnamaldehyde	2 mg/mL *S.* Typhimurium	Reduced biofilm population by 6 log CFU/cm^2^	[85]
Carvacrol	0.66 mM *P. carotovorum*	Reduced EPS synthesis	[88]

## Data Availability

Data from the reviewed articles can be found in Pubmed (https://pubmed.ncbi.nlm.nih.gov/, accessed on 1 September 2021) and Scielo (https://scielo.org/es/ accessed on 1 September 2021).
